# Perinatal compromise affects development, form, and function of the hippocampus part two; preclinical studies

**DOI:** 10.1038/s41390-024-03144-0

**Published:** 2024-03-22

**Authors:** Tegan A. White, Suzanne L. Miller, Amy E. Sutherland, Beth J. Allison, Emily J. Camm

**Affiliations:** 1 The Ritchie Centre, Hudson Institute of Medical Research, Clayton, VIC Australia; 2 Department of Obstetrics and Gynaecology, Monash University, Clayton, VIC Australia

## Abstract

**Abstract:**

The hippocampus is a vital brain structure deep in the medial temporal lobe that mediates a range of functions encompassing emotional regulation, learning, memory, and cognition. Hippocampal development is exquisitely sensitive to perturbations and adverse conditions during pregnancy and at birth, including preterm birth, fetal growth restriction (FGR), acute hypoxic–ischaemic encephalopathy (HIE), and intrauterine inflammation. Disruptions to hippocampal development due to these conditions can have long-lasting functional impacts. Here, we discuss a range of preclinical models of prematurity and FGR and conditions that induce hypoxia and inflammation, which have been critical in elucidating the underlying mechanisms and cellular and subcellular structures implicated in hippocampal dysfunction. Finally, we discuss potential therapeutic targets to reduce the burden of these perinatal insults on the developing hippocampus.

**Impact:**

- The review explores the preclinical literature examining the association between pregnancy and birth complications, and hippocampal form and function.
- The developmental processes and cellular mechanisms that are disrupted within the hippocampus following perinatal compromise are described, and potential therapeutic targets are discussed.

## Introduction

The hippocampus is a vital brain structure that plays essential roles in emotional regulation, learning, memory and cognitive functions. This small brain region has a complex developmental profile that begins very early in gestation and continues through adulthood, with a peak developmental growth spurt occurring in the latter period of gestation and into neonatal life. Thus, hippocampal development overlaps with serious pregnancy complications, including preterm birth, fetal growth restriction (FGR), acute hypoxic-ischaemic insult at birth and intrauterine inflammation. Both clinical and preclinical research have provided links between perinatal insult, altered hippocampal structure, and adverse neurodevelopmental consequences, including working memory deficits and poor cognitive outcomes. To further examine cellular vulnerability, preclinical animal studies of pregnancy and birth complications have investigated hippocampal cellular development and shown that the pyramidal neurons of the hippocampus are susceptible to perinatal compromise.^1–4^

Part one of this review^5^ summarised hippocampal development and discussed evidence from human studies showing that common perinatal insults can disrupt hippocampal development, form, and function. In part two of this review, we focus on preclinical studies and their vital use in providing critical insights regarding cellular structural changes within the hippocampus in response to these common perinatal insults. Preclinical studies are imperative in this research, allowing a depth of knowledge not available in the clinical setting, with the capacity to reveal the mechanisms driving hippocampal dysfunction. Finally, we discuss critical knowledge gaps in the field, particularly regarding efficacious treatment options to prevent or ameliorate hippocampal injury following complications of pregnancy.

### Overview of hippocampal structure

**Fig. 1:**
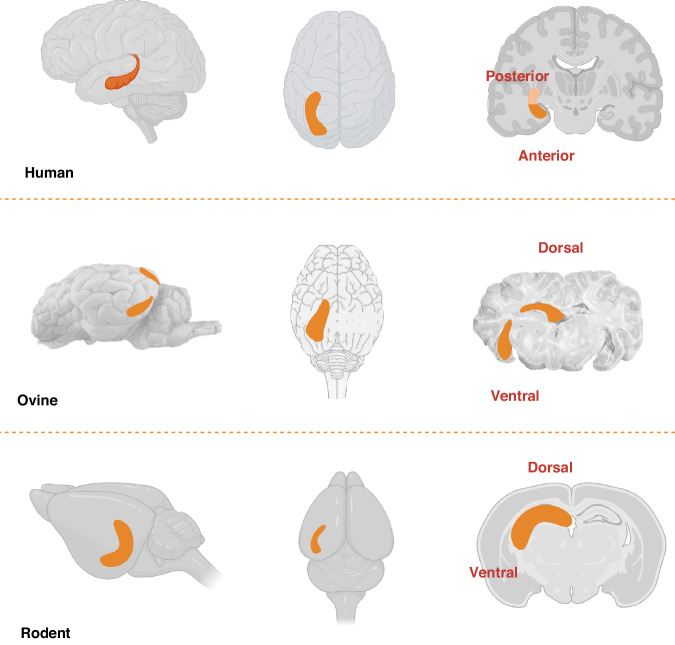
Representative diagrams of human, ovine, and rodent hippocampus depicting the dichotomous dorsal/posterior and ventral/anterior regions of the hippocampus. Image created with BioRender.com (agreement number TA25Z7FMRM).

The *hippocampal formation* comprises four cornu ammonis (CA) fields, CA1–CA4, dentate gyrus (DG), subiculum and entorhinal cortex (EC). The intricate structure of the hippocampus is detailed in part one of this review.^5^ The distinct shape of the hippocampus is divided into dorsal and ventral horns, evident in the ovine and rodent brains, which correspond to the posterior and anterior hippocampus in humans (Fig. 1^6,7^). The longitudinal axis gives rise to specific hippocampal circuits with defined functions; the dorsal/posterior hippocampus is linked to cognitive processing and spatial memory, whereas the ventral/anterior hippocampus regulates emotional processing and responses.^8–10^ The concept of dichotomous dorsal/posterior and ventral/anterior regions of the hippocampus having independent connectivity is widely accepted. However, there remains a question of whether these two regions are functionally distinct or if the *hippocampal formation* works as an integrated structure.^9,11^

Whilst pyramidal neurons are considered the most important cell within the hippocampus with extensive roles in overall hippocampal function and connectivity,^12^ there are many other supporting cells and subcellular factors that are essential for optimal hippocampal function and may be impacted by perinatal compromise. As will be discussed in this review, growth factors and signalling pathways may also be impaired by disruptions to development.^13–16^ One advantage of preclinical studies is the ability to determine the in-depth and detailed microstructure of cells, and these findings have been used in a neuroscience context to significantly advance the field. Characterising the mechanisms that contribute to hippocampal vulnerability has been hampered by the difficulty of quantifying histopathology and pathological processes within the human hippocampus, with the exception of MRI studies that have assessed gross brain and hippocampal volumes. Here, preclinical research can address this knowledge gap to provide cellular-level insight into disrupted hippocampal development, and potential mechanisms of injury, in response to perinatal challenges. Preclinical studies allow assessment of neuropathology from gross morphology through to the subcellular microscopic level and determination of structure–function relationships. Revealing details of the hippocampus at this intricate level is only possible with preclinical models and enhances our understanding of this brain region and its vulnerability to conditions such as preterm birth, FGR, acute hypoxic–ischaemic insult at birth, and intrauterine inflammation. With this knowledge, targeted therapies can be pursued with the aim of lasting improvements for children affected by perinatal compromise.

### Preclinical studies of perinatal compromise

**Fig. 2:**
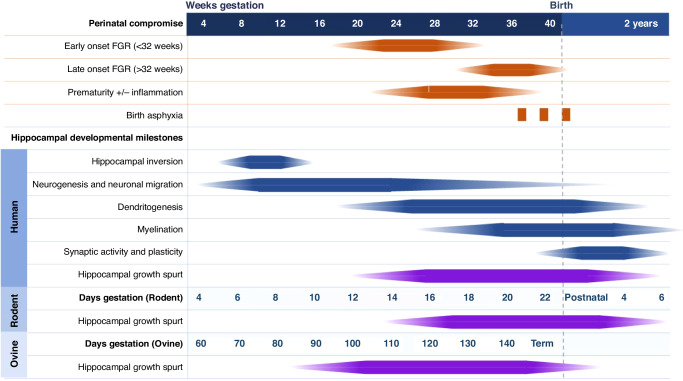
Timeline of typical points of perinatal injury against a timeline of hippocampal development with gestation milestones for human, rodent and ovine. Dark solid colour indicates peak development or time of insult.

The cellular origins of hippocampal vulnerability to injury and long-term neuropathology in the hippocampus are not fully elucidated. What is clear, however, from data presented in Part 1 of this Review,^5^ is that disruptions to hippocampal growth that occur during pregnancy and at birth persist for a lifetime, evident as reduced volume and altered function. The timing of hippocampal development relative to gestation described in Part 1 of this review,^5^ has been expanded in this review to include ovine and rodent timelines relative to human gestation milestones (Fig. 2). The hippocampal growth spurt describes the period when this region is undergoing its maximal rate of development and increase in physical dimension, commencing when neurogenesis is complete and predominantly contributed by the outgrowth of neuronal processes (dendritogenesis), glial cell proliferation and myelination.^17^ For the human brain, this growth spurt occurs from approximately mid-gestation through to infancy,^18,19^ and this is mirrored in the specific development of hippocampal neurons.^20^ Unfortunately, this hippocampal growth spurt period coincides with many perinatal compromise situations of premature birth, FGR and hypoxic episodes (Fig. 2).

Preclinical animal models have been used to reveal the mechanisms driving hippocampal dysfunction in pregnancy complications, as shown in baboons, sheep, rabbits, guinea pigs, rats, mice, and chicken embryos. The effects of human preterm birth are difficult to model since the physiological mediators of human parturition are unique and multifactorial, and the endocrine cascade that initiates and controls parturition differs between species.^21^ However, the effects of prematurity per se on the developing hippocampus can be examined in species with a similar litter size, such as baboons and sheep, with offspring delivered early either by induced preterm labour or delivered via caesarean surgery.^22,23^ Intrauterine inflammation often goes hand-in-hand with prematurity and can be induced in preclinical models with the administration of bacterial toxins or cytokines (e.g. LPS or IL-1β).^24–27^ FGR can be modelled in a myriad of ways, from *in utero* surgical manipulation of uterine or umbilical arteries, to maternal undernourishment and infusions of L-NAME, thromboxane A2-analogue (TXA) and dexamethasone to reduce fetal growth.^2,13,16,28–30^ Conditions of hypoxia are often either surgically induced with the occlusion of umbilical arteries or via maternal or environmental hypoxia. Therefore, there is an ability to modify the severity and length of the insult to mimic severe insults of birth asphyxia or moderate long-term hypoxic exposure.^1^ The duration, timing and severity of insult can be examined separately in animal studies, in turn altering the degree to which the *hippocampal formation* structure and function is disrupted, giving insight into the clinical consequences of these conditions.

Given the focus of the current review is on hippocampal neuropathology, one needs to consider when the insult or treatment is initiated relative to the status of developmental processes (e.g. neurogenesis, migration, proliferation or myelination). The sequence of key events in hippocampal development is largely consistent between mammalian species^31^ (Fig. 2). However, the period and complexity of these developmental processes differ between species. For example, brain development extends postnatally in rodents, which is a consideration when inducing conditions of in-utero compromise and translating outcomes to human stages of brain development.^21,32^ Whereas guinea pigs are precocial brain developers allowing for manipulation of the in-utero environment to mimic conditions of perinatal compromise.^21,32^ Rabbits are increasingly incorporated into studies of fetal development as timelines of lung and brain development are relatively similar to humans.^33^ Whilst there are practical (size) challenges to using sheep, the similarities in brain size and structure to the human brain, including the gyrencephalic cortex, provide advantages.^34^ The most analogous model to human development, however, is non-human primates due to the phylogenetic proximity of baboons and humans and key similarities in brain structure and function.^21,35^ Consideration of the strengths and weaknesses of any animal model of perinatal compromise is fundamental to effective and meaningful translation of preclinical research.

Table 1 summarises a select variety of seminal studies to demonstrate the breadth of disruptions to hippocampal cellular structure that occur in response to perinatal compromise whilst acknowledging that this is not an exhaustive list of every preclinical study conducted.

**Table 1:** Preclinical studies investigating the impact of perinatal compromise on the hippocampus.

Species and model	Manipulation	Age at study	Hippocampal morphology	Hippocampal function	Reference
Baboon (Prematurity)	Preterm baboons born 125, 140 and 160dGA (term 185dGA).	Tissue collected after delivery at 125, 140 and 160dGA.	Cell loss was observed in the CA2/3 region of the hippocampus, and reactive astrogliosis was observed.	NA	Inder et al.^37^
Rabbits (Prematurity)	Preterm rabbits were born on embryonic day 28.5 (term 32 days).	Preterm rabbits were assessed at day 28–30, and tissue collected at day 30.	↓ dendritic arborisation and spine density in hippocampal CA1.	↓ social and object novelty ↓ recognition and memory ↑ anxiety behaviour	Klebe et al.^22^
Rabbits (Prematurity)	Preterm rabbits born postconceptional age 28 days (term 32 days).	Behavioural assessments at postconceptional age 32 days, brain tissue collected at 32 days.	↓ neuron density, oligodendrocyte pre-cursor proportion in the hippocampus. ↑ pyknotic and apoptotic cells, evidence of astrogliosis	Significant motor deficit, less pain responsive.	van der Merwe et al.^38^
Guinea Pigs (Prematurity)	Preterm pups born 62dGA (term 69dGA) were treated with ganaxolone.	Ganaxolone 2.5 mg/kg twice daily until term equivalence. Behavioural testing at day 25, and tissue collected at day 28.	↓ myelination in the CA1 region of the hippocampus in premature non-treated animals.	↑ activity in premature non-treated animals and disinhibited social response.	Shaw et al.^39^
Sheep (FGR)	Chronic placental insufficiency induced by umbilicoplacental embolization	Surgery at 115dGA, tissue collected at 140dGA (term ~147 days).	↓ BDNF protein expression in the hippocampus.	NA	Duncan et al.^16^
Rabbits (FGR)	Ligation of 40–50% of uteroplacental vessels.	Surgery at 25dGA, born at 30 days (term ~31 days). A neurobehavioural assessment was completed then tissue was collected on postnatal day 1.	↓ fractional anisotropy in FGR hippocampus. Correlations between hippocampus and neurobehavioural domains.	↓ neurobehavioural performance (motor activity and olfactory function—social interaction)	Eixarch et al.^52^
Guinea Pigs (FGR)	FGR surgery and prenatal stress (PS).	Stress protocol commenced at 40dGA. Tissue taken at 69dGA (term ~71 days).	↓ myelin basic protein coverage in the hippocampus in FGR and FGR + PS males.	NA	Cumberland et al.^2^
Guinea Pigs (FGR)	Chronic placental insufficiency induced by unilateral uterine artery ligation.	Surgery was at 28–30dGA, fetus was collected for tissue analysis at 60dGA (term ~71 days).	↓ dendritic complexity of CA1 neurons in growth-restricted fetuses	NA	Dieni et al.^14^
Guinea Pigs (FGR)	Chronic placental insufficiency induced by unilateral uterine artery ligation.	Surgery was at 28–30dGA, fetus was collected for tissue analysis at 60dGA (term ~71 days).	↓ BDNF protein expression in hippocampus ↑ TrkB protein expression	NA	Dieni et al.^15^
Guinea Pigs (FGR)	Maternal nutrient restriction	Guinea pigs are fed 70% of the control diet before pregnancy up to mid-pregnancy. Tissue collected from near-term fetuses	↑ hypoxia (HP-1) and oxidative stress markers in the hippocampus	NA	Maki et al.^86^
Rats (FGR)	L-NAME induced FGR (50 mg/kg/d), control FGR and glutathione-treated FGR.	L-NAME intervention 9–19dGA. Glutathione was administered either postnatal day 4–9 or 25–31.	↓ corrected total cell fluorescence VGLUT1 in the hippocampus in FGR, treatment of glutathione caused ↑	↓ in spontaneous alternation percentage (SAP), early treatment with glutathione increased SAP.	Shallie et al.^30^
Mice (FGR)	Mice inserted with micro-osmotic pump infusion of thromboxane A2-analogue (TXA) to induce FGR	Pump inserted at embryonic day 12. Tissue collected at embryonic day 15.5 or 19, or on postnatal day 18 or 40	↓ hippocampal volumes in IUGR mice Accelerated embryonic DG neurogenesis and Sox2^+^ neural stem cell depletion	↓ short-term adult learning and memory deficits (novel object recognition and fear conditioning)	Brown et al.^28^
Mice (FGR)	Dexamethasone-induced FGR and protein restriction-influenced FGR mouse models.	Behavioural experiments were conducted at 8 to 12 weeks of age.	↓ hippocampal neurogenesis with FGR. ↓ proliferations of neuronal stem cells. ↓ Tet1 protein expression.	Impaired learning and memory in FGR offspring (novel object recognition and Morris water maze)	Chen et al.^13^
Mice (FGR)	Mice inserted with micro-osmotic pump infusion of thromboxane A2-analogue (TXA) to induce FGR	Pump inserted at embryonic day 12. Tissue was collected at postnatal day 10, 18 and 40.	Hippocampal synaptic plasticity disturbed with FGR balance between excitatory and inhibitory neuronal development impaired.	NA	St. Pierre et al.^29^
Rats (FGR)	FGR induced by a low-protein diet or bilateral uterine ligation.	18 dGA surgery performed. Assessed at postnatal day 1, 12 and 180.	mTOR signalling in the hippocampus dysregulated with FGR	NA	Schömig et al.^49^
Sheep (Hypoxia)	Umbilical cord occlusion (UCO) until MAP decreased to 18–20mmHG—severe asphyxia	Lambs delivered at 139–141dGA, UCO, with clamp-on delivery. Lambs were resuscitated and maintained for 72 h.	Neuronal degeneration in hippocampus ↑ astrogliosis in CA1 of the hippocampus	NA	Aridas et al.^61^
Sheep (Hypoxia)	UCO for 25 min—severe asphyxia	Instrumentation surgery on 98–100dGA fetal sheep, experimentation 4–6 days after. Fetuses recovered for 3-, 7-, 14- and 21-days post UCO, before post-mortem.	↓ hippocampal total area with UCO ↓ neuronal density ↑ microglia and astrocyte activation in the hippocampus	NA	Lear et al.^54^
Sheep (Hypoxia)	UCO for 10 min—severe asphyxia.	Instrumentation surgery on 120–127dGA fetal sheep, experimentation 72 h after.	Neuronal loss in the hippocampus of the experimental group, more neurons were lost in the dorsal horn than ventral.	NA	Mallard et al.^55^
Sheep (Hypoxia)	Transient hypoxia-ischaemia (HI) and hypoxia (Hx) in a preterm fetal sheep model.	88–92dGA (0.65 gestation) surgery performed, HI studies 3 days later.	↓ hippocampal volume in HI and Hx. ↓ complexity, CA1 maturation augmented.	Long-term synaptic potentiation is impaired following short-term hypoxia.	McClendon et al.^1^
Sheep (Hypoxia	UCO for 10 min—severe asphyxia.	Instrumentation surgery on 126dGA fetal sheep, UCO at 130dGA, post-mortem conducted 48 h after.	↑ pyknotic cells in the CA1 region of hippocampus ↑ astrocytes in the CA1 region of hippocampus ↑ cellular lipid peroxidation	NA	Yawno et al.^56^
Rats (Hypoxia)	Intermittent asphyxia at 9 and 5% O_2_ at postnatal day 11 rat pups.	Asphyxia at postnatal day 11. Behavioural tests from age 3–14 months.	Neurodegeneration in hippocampus and thalamus in post-asphyxia rats.	Impaired spatial learning and memory and increased anxiety.	Gailus et al.^64^
Mice (Hypoxia)	Chronic sublethal hypoxia (CSH).	Hypoxia from postnatal day 3 to day 11. DTI at days 15, 17, 38, 45 and 51.	↓ connectivity in CSH mice, damage to axons and disturbance to development.	Hyperactivity observed in CSH mice, impaired performance on spatial memory tasks.	Chahboune et al.^3^
Chicken Embryos (Hypoxia)	Hypoxic insult for 24 h followed by reoxygenation period and growth hormone treatment.	Chicken embryos at 15 days embryogenesis.	Growth hormone can cross the BBB and provide hippocampal neuroprotection to hypoxia.	NA	Baltazar-Lara et al.^76^
Sheep (Inflammation)	Preterm fetal sheep model exposed to LPS	Intra-amniotic injection of LPS at 117 days gestation	↑ cell death in the hippocampus	NA	Yawno et al.^25^
Sheep (Inflammation)	Preterm fetal sheep model exposed to LPS	LPS IV injection on days 109, 110 and 111 of gestation	↑ activated microglia in the hippocampus	NA	Yawno et al.^68^
Rats (Inflammation)	Fetal rats exposed to intra-amniotic LPS.	LPS administered 2 days before birth, and tissue was taken at postnatal day 7.	Activation of microglia in the hippocampus of LPS rats.	NA	Gisslen et al.^27^
Mice (Inflammation)	Intraperitoneal LPS injections.	Postnatal day 14, behavioural testing was conducted at 8–9 weeks old.	↓ hippocampal volume in the LPS group.	Memory deficits in LPS mice, impaired retrieval and retention of hippocampus-dependent learning.	Malaeb et al.^67^
Mice (Inflammation)	Intraperitoneal IL-1β injections	IL-1β administered twice daily from postnatal day 1–4. Tissue was collected at postnatal days 2, 5 and 10, and the long-term cohort conducted behavioural testing up to postnatal day 76.	↑ pro-inflammatory cytokines and chemokines in the hippocampus ↓ growth of hippocampal neural progenitors	↑ anxiety-like behaviours (elevated plus maze and open field test) ↓ spatial memory function (Barnes maze task)	Veerasammy et al.^26^

*CSH* chronic sublethal hypoxia, *dGA* days gestational age, *DTI* diffusion tensor imaging, *FGR* fetal growth restriction, *GA* gestational age, *GM-IVH* germinal matrix-intraventricular haemorrhage, *HP-1* hypoxyprobe-1, *IL-1β* interleukin-1 beta, *IVH* intraventricular haemorrhage, *L-NAM* Nω-nitro-l-arginine methyl, *LPS* lipopolysaccharide, *MAP* mean arterial pressure, *MRI* magnetic resonance imaging, *mTOR* mammalian target of rapamycin, *NA* not assessed, *Tet1* ten–eleven translocation methylcytosine dioxygenase 1, *TrkB* Tropomyosin receptor kinase B, *UCB-MSCs* umbilical cord blood-mesenchymal stem cells, *UCO* umbilical cord occlusion, *VLBW* very low birth weight, *VPT* very preterm.

### Preclinical studies of prematurity

Preterm birth has a wide range of implications for the affected infant, with the particular vulnerability of the brain as development typically continues throughout gestation.^36^ In preclinical studies of preterm birth and insults to the preterm brain, altered hippocampal structure is commonly observed. Inder et al.^37^ examined brain microstructure using a combination of MRI and histopathology in baboons delivered preterm at 0.78 gestation (~26 weeks of human brain development) and followed to term-equivalent age. This study found significant neuronal cell loss in the CA2/CA3 regions of the hippocampus of premature baboons with accompanying reactive astrogliosis.^37^ Studies in rabbits comparing the effects of preterm birth (0.87 gestation) with term birth, followed up for one month after term-equivalent age, showed that hippocampal CA1 neuron density and dendritic complexity were reduced, oligodendrocyte precursor population was reduced, together with an increase in pyknotic and apoptotic cells in the preterm brains.^22,38^ A similar study in guinea pigs found that myelination was reduced in the hippocampus of prematurely born animals.^39^ The works by Klebe et al.^22^ and Shaw et al.^39^ followed up with functional testing and showed that premature birth was associated with altered social response, reduced memory capacity, and an increase in activity and anxiety behaviours that are consistent with the clinical presentation of children born preterm and demonstrating signs of ADHD and ASD.^40^

In comparison to the wealth of clinical studies investigating the impact of preterm birth and the preclinical studies of other conditions of perinatal compromise, the preclinical literature on prematurity is relatively sparse. As noted above, it is difficult to model the complexities of preterm birth (both causes and consequences) in animal models. However, animal studies do allow the separation of confounding factors.

### Preclinical studies of fetal growth restriction

FGR is a complex condition with varied aetiology and progression.^41^ FGR is most often caused by suboptimal placental function, termed placental insufficiency, which causes chronic fetal hypoxia and hypoglycaemia.^42^ The impact of FGR on the developing brain heavily depends on the timing of FGR (early- or late-onset), duration and severity of fetal hypoxia, and gestational age at birth.^43^ Preclinical animal models of FGR have been used to examine the vulnerability of the hippocampus. A 12-h period of placental insufficiency, induced by a vascular clamp on the maternal common internal iliac artery, in 0.6 gestation fetal sheep (equivalent to ~26 weeks of human brain development) caused a >30% decrease in the density of CA1 neurons and concomitant increase in astrocytes in the ventral hippocampus at histological examination 35 days after the insult.^44^ Moreover, in fetal sheep, late-onset placental insufficiency (0.7 gestation) induced by single umbilical artery ligation, resulting in chronic fetal hypoxia and hypoglycaemia, was associated with a greater proportion of CA3 hippocampal neurons with an abnormal morphological appearance but without overt cell loss in the CA1 or CA3 regions.^45^ Using the same ovine model of late-onset placental insufficiency, cellular apoptosis and oxidative stress were upregulated within the FGR hippocampus compared to control,^46^ and basal blood flow to the hippocampus was relatively low compared to other neuron-rich grey matter regions.^47^ In ovine FGR fetuses, blood flow to the hippocampus was reduced by 50% compared to control animals, while it was relatively spared in the brainstem.^47^ This data supports earlier studies in FGR guinea pigs.^48^

In addition to hippocampal cell damage being reported in preclinical studies of pregnancy compromise, other studies have shown reduced hippocampal neurogenesis together with disruptions to myelination.^2,13,29,30,49^ In growth-restricted guinea pigs and fetal sheep, the concentration of brain-derived neurotrophic factor (BDNF) was reduced in the hippocampus^14–16^ with a concomitant reduction in dendritic complexity of CA1 pyramidal cells.^15^ This is an important observation, as BDNF is an essential growth factor for dendritic outgrowth.^50^ Conversely, a study comparing adolescent rodents born growth restricted and control offspring reported no difference in hippocampal levels of the mature BDNF isoform and no difference in neuronal morphology of DG cells,^51^ suggesting potential attenuation of these deficits with ageing. An additional study in protein-restricted, growth-restricted rats revealed critical cellular and molecular mechanisms of FGR-induced deficits, noting the loss of ten-eleven translocation (Tet) protein (Tet1) and DNA hypermethylation of Notch signalling genes.^13^ This, in turn, caused a downstream reduction of neural stem cell (NSC) proliferation, correlated with deficits in learning and memory.^13^ Interestingly, cell death within the CA1 cells was not observed in FGR offspring, which prompted the authors to propose that the deficits in the hippocampus likely arose from reduced NSC proliferation and not an increase in hippocampal neural cell death.^13^ This is further supported by Brown et al.,^28^ where DG vulnerability and subsequent NSC depletion and premature neurogenesis were observed in postnatal FGR guinea pigs. Another potential mechanism underlying FGR-induced hippocampal deficits is the altered mammalian target of rapamycin (mTOR) signalling in the central nervous system. The mTOR pathway is an essential cell signalling pathway and plays an important role in brain development, specifically cellular growth and metabolism.^49^ To investigate mTOR signalling in the context of FGR, Schömig et al.^49^ induced FGR in rodents via either a maternal low-protein diet or intrauterine surgical stress. The results demonstrated that mTOR signalling was differentially dysregulated depending on the underlying cause of FGR, reflecting the complex heterogenous nature of growth restriction. In growth-restricted rabbits, Eixarch and colleagues^52^ used brain MRI to demonstrate brain reorganisation within the hippocampus, which was significantly associated with alterations in neurobehavioural assessments, highlighting the direct functional outcomes of impaired hippocampal development.

### Preclinical studies of hypoxic–ischaemia

Episodes of hypoxia–ischaemia (HI) reportedly occur more frequently in the preterm period than at term.^53^ The effects of hypoxic-ischaemic insults can be induced *in utero* (in sheep) to examine the specific effects on the preterm brain. Lear et al.^54^ induced a severe, acute HI insult in preterm fetal sheep (0.7 gestation, equivalent to human brain development at ~28–32 weeks gestation) and assessed the progression of hippocampal neuropathology over a 21-day time course in CA subfields and the DG. This study showed that within 3 days of HI insult, there was an increase in both astrocyte and microglial cell numbers in the CA3 region, with reduced neuronal cell numbers, and at 14 days post-insult, the hippocampal area was reduced.^54^ Assessment of the relative vulnerability of the hippocampal neuronal populations across the subfields CA1/2, CA3, CA4 and DG revealed that all regions were susceptible to cell death.^54^ A study in preterm fetal sheep at 0.65 gestations compared the effects of transient hypoxia–ischaemia, induced by brachiocephalic artery occlusion, to hypoxia alone and found that estimated hippocampal volume was reduced 4 weeks after insult in response to both occlusion and hypoxia. However, volume deficits were not caused by neuronal cell loss per se but rather were mediated by an altered developmental profile of basal and apical dendritic arborisation.^1^ Regarding the potential mechanisms of altered neuronal development, McClendon and colleagues found a significant association between the degree of dendritic arborisation, fetal systemic hypoxaemia, and metabolic stress with the altered profile of CA1 neurons linked to a reduction in glutamate release.^1^

The cellular effects of perinatal (birth) asphyxia on the term-equivalent brain have also been characterised in preclinical animal studies. Interestingly, at term-equivalent age, multiple studies demonstrate that hippocampal neurons demonstrate significantly greater susceptibility to acute hypoxic–ischaemia compared to other neurons. This was first documented by Mallard et al.,^55^ who demonstrated in fetal sheep at term-equivalent brain age exposed to an acute asphyxic episode (10 min of umbilical cord occlusion), that hippocampal neurons are more susceptible to cell death than any other population of neurons studied (striatum, dentate gyrus, thalamus, lateral cortex, and amygdala). In fetal sheep exposed to umbilical cord occlusion at 0.89 gestation, it was found that hippocampal CA1 neurons showed high levels of pyknosis, in excess of other brain regions examined, but did not show evidence of caspase-3 mediated cell death (where other brain regions did).^56^ Yawno and colleagues^56^ also demonstrated that astrogliosis was induced by acute hypoxia in the hippocampus, and the hippocampus showed the highest levels of cellular lipid peroxidation, but there was no alteration in the density of microglial cells.^56^ Similarly, work by Gunn and colleagues has consistently demonstrated that an acute hypoxic–ischaemic insult in term-equivalent fetal sheep induces hippocampal damage, comprising neuronal cell loss and suppression of microglia.^57–59^ Ginet et al. examined cell death pathways in CA1 and CA3 populations of cells in neonatal rats exposed to a severe hypoxic-ischaemic insult and found that cell death predominantly occurred via autophagic rather than apoptotic mechanisms.^60^ Ginet^60^ also showed striking differences in the profile of cell death across hippocampal subfield regions, with caspase-3-mediated apoptosis confined to CA1 neurons, whilst autophagic cell death was evident in CA3 neurons.^60^ When acute severe asphyxia is induced at birth in lambs, and the lamb is then immediately delivered and maintained in a neonatal care environment for 72 h, the hippocampus is highly vulnerable to both apoptotic and necrotic cell death, with a similar degree of injury observed in the CA1 and DG regions.^61–63^ It is worth noting that when birth asphyxia is severe, as in the Aridas studies,^61–63^ hippocampal neurons do not demonstrate preferential susceptibility to cell death in comparison to other neuron-rich areas, including the cortex and thalamus, with hippocampal sub-regions comparable broadly with all regions to show increased astrogliosis, microglial activation, and indices of oxidative stress.^62^ Finally, a study in neonatal rats that set out to examine the long-term consequences of severe asphyxia at postnatal day 11 on regional differences in hippocampal morphology showed that both the dorsal and ventral hippocampus were similarly affected.^64^ The CA3 and dentate hilus neurons showed neurodegeneration, whereas CA1 neurons were relatively spared.^64^ Critically, the Gailus et al.^64^ study was also able to correlate neuronal loss in CA3 dorsal hippocampal cells with deficits in spatial learning, while neuropathology in the ventral hippocampus reflected an increase in anxiety-like behaviours in adult rats reflecting the dichotomous neural pathways in the hippocampus.

Moreover, preclinical studies reveal that there are differences in the capacity of hippocampal neurons to withstand perinatal compromise in the preterm versus the term-equivalent brain, with cell loss more likely to occur in the term-equivalent fetus. This is not surprising, given that the preterm fetus is able to mount a very effective response to moderate hypoxia and can tolerate hypoxia for a longer period without sustaining overt brain injury compared to the fetus at term.^53^ In the preterm period, a transient period of moderate hypoxia did not tend to result in widespread hippocampal cell death but rather impaired dendritogenesis and arborisation, thereby reducing hippocampal volume.^1^ In contrast, transient moderate hypoxia at term-equivalent age resulted in selective loss of hippocampal neurons where other grey matter regions were spared.^55^ More severe hypoxic or asphyxic insult was shown to cause widespread neuronal cell loss in the hippocampus and other grey matter regions.^61,62,65^ Comparing an acute severe hypoxic episode in the 0.7 gestation sheep fetus^54^ versus the term fetus,^63^ with brain collection three days later shows that the neuronal degeneration in the hippocampus is present to a similar degree compared to the cortex, and oxidative stress and neuroinflammation are evident in the hippocampus of both the preterm and term brain. Whether the perinatal insult is caused by hypoxia or inflammation does not appear to have a strong impact; for example, in LPS-exposed preterm fetal sheep, that was sufficient to induce moderate hypoxaemia, pro-inflammatory cytokine response and white matter damage, there was no evidence of neuronal injury or astrogliosis in the hippocampus.^66^ Combined, these studies indicate that the hippocampus in the preterm brain is relatively more resilient than in the term brain, however, at term age, hippocampal neurons demonstrate an increased susceptibility to cell death than other grey matter neuron populations.

### Preclinical studies of intrauterine inflammation

Intrauterine inflammation/chorioamnionitis is a common cause of preterm birth.^24^ Preclinical studies have utilised *in utero* administration of lipopolysaccharide (LPS), a bacterial toxin, to induce preterm neuropathology.^25,27,67,68^ This is demonstrated in fetal sheep studies where LPS administration at 0.7 gestation, either via intravenous (150 ng/kg^68^) or intra-amniotic injection (20 mg^25^), caused an increase in cell death and activation of microglia in CA1 and CA3 regions.^25,68^ LPS administration to postnatal day 3–13 mice (0.3 mg/kg), equivalent to late preterm human brain development, resulting in a 15–20% reduction of total hippocampal volume compared to controls, with associated deficits in working memory.^67^ Interleukin-1β (IL-1β) is a potent pro-inflammatory cytokine produced during *in utero* inflammation and can be utilised preclinically to investigate perinatal inflammation. Veerasammy et al.^26^ administered IL-1β (10 ng/gm intraperitoneal) to mice on postnatal days 1–5 (~28–32 weeks human brain development). Pro-inflammatory cytokines and chemokines in the hippocampus were elevated within one day following exposure and were associated with anxiety-like behaviours and impairments in spatial memory.^26^ These studies highlight the susceptibility of the hippocampus to a third-trimester inflammatory insult and the potential for long-term negative functional consequences.^26^ In their comprehensive literature review, Green and Nolan^69^ detailed consistent adverse effects of inflammation, induced by multiple immunogens, on the developing hippocampus, with alterations in hippocampal neurogenesis, neuronal outgrowth, and neuronal cytoarchitecture across hippocampal subregions, with life-long consequences for behaviour.

Taken together, it is evident that adverse perinatal insults have significant effects on the developing hippocampus. However, preclinical studies showed that only the most severe hypoxic-ischaemic insults caused overt neuronal loss in the hippocampus in both the preterm brain and the term brain.^55,56^ Mild to moderate insults, such as preterm birth and/or FGR, did not result in hippocampal cell death but caused altered dendrite outgrowth and patterns of arborisation,^1^ which resulted in altered patterns of connectivity and functional deficits.^41^ Accordingly, it can be said that the most striking and consistent finding is decreased volume of the hippocampus, caused by interruption of dendritogenesis and abnormal neuronal morphology, and this is also reflected in clinical findings.^5^

### Future directions

This review provides additional depth to our understanding of the vulnerability of the hippocampus to perinatal compromise by describing cellular and subcellular mechanisms driving key hippocampal processes. Preclinical studies, in particular, suggest that hippocampal vulnerability arises due to the density of highly metabolic neurons and relatively low basal blood flow to the hippocampus, which is further exacerbated in response to a hypoxic insult where, in contrast, other grey matter regions may be relatively spared.^47,70^ Critically, the timing of common pregnancy complications in the final trimester of pregnancy has a profound adverse impact on hippocampal dendritic outgrowth and synaptic connections which are undergoing peak development during this period, resulting in a reduction in hippocampal volume.

Interestingly, the preclinical studies presented also reveal that there are subtle differences in the relative susceptibility of hippocampal neurons when comparing hypoxic-ischaemic insults in the preterm and term-equivalent period, with a brief moderate hypoxic episode causing selective neuron loss in the hippocampus of term but not preterm brains. FGR is not associated with overt neuronal cell loss in the hippocampus but is associated with a decrease in neurotrophic factors such as BDNF^14,16^ and a reduction in the proliferative pool of NSCs.^13^ A common finding across all perinatal conditions is that altered hippocampal neuronal development occurred in parallel with regional astrogliosis,^37,38^ and to a lesser extent with microglial cell activation.

Within the subregions of the *hippocampal formation*, the findings presented here suggest both structural and functional differences in neuronal response to perinatal compromise. Histological studies show that regions in the cornu ammonis (CA1 to CA4) and DG are distinguishable due to their morphological variability and connectivity. Preclinical studies in which multiple *hippocampal formation* subfields have been assessed demonstrate that discrete differences exist in subfield neuron response vulnerability to injury.^45,54,55^ The effects of FGR,^45^ and acute hypoxic–ischaemic insult in both the preterm^54^ and the term-equivalent brain,^55^ have shown that neurons within the CA3 region have an increased susceptibility to cell death than CA1 neurons in preclinical models of disease. Lear and colleagues showed that neurodegeneration in the CA3 field occurred more consistently across the various time points studied than other CA and DG regions, while CA4 neurons were relatively spared.^54^ The reason for varied susceptibility to insult across the hippocampal subfields is likely to be due to a number of factors, with Ginet and colleagues finding that cell death via apoptosis was more likely to occur in CA1 neurons while CA3 neurons responded to hypoxic–ischaemic insult with autophagy.^60^ Cellular metabolic factors and distribution of local vasculature and blood flow supply are also likely to mediate response to injury, with Mallard et al.^55^ finding that cell death in CA3 neurons was correlated with the degree of fetal hypotension. There is limited literature linking subregional injury to distinct functional implications, as detecting such region-specific differences while other regions are spared would require lesions of CA1 or CA3 cell populations,^71,72^ which has not been modelled for pregnancy compromise. In adult studies, subregional lesions have revealed both CA1 and CA3 regions play a key role in memory encoding while CA1 cells are more specific for memory retrieval.^71^ We found that it was not common for researchers to document the area of hippocampal study across the longitudinal axis; that is, the dorsal/posterior hippocampus and ventral/anterior hippocampus. One study undertaken in rats showed that neuronal degeneration was present in the dorsal hippocampus of post-asphyxial rats and was linked to a decline in spatial learning, a skill attributed to the dorsal hippocampus.^64^ The possible dichotomy between these regions is very interesting, and differential vulnerability to each of the pregnancy and birth complications may shed light on the cognitive and behavioural dysfunctions that are associated with these conditions.

The preclinical studies presented here provide an opportunity to examine common causal pathways that may contribute to hippocampal injury or altered development. Multiple studies across all perinatal complications noted that astrogliosis was present alongside hippocampal injury, and to a lesser extent, microgliosis and oxidative stress were also observed. Interestingly, the expression growth factor BDNF was reduced in association with impaired hippocampal structure.^14,16^ These findings provide an opportunity to explore therapeutic interventions. A study in rats in which FGR was induced with maternal dexamethasone administration treated a cohort of animals with maternal lactoferrin supplementation and showed that lactoferrin treatment restored BDNF levels and neuronal cell number within the hippocampus.^73^ BDNF is a critical neurotrophic modulator of dendrite outgrowth, including for hippocampal neurons,^74^ and is also neuroprotective when administered to the immature brain in response to a hypoxic-ischaemic insult.^75^ It is, however, interesting to note that in mature hippocampal neurons, the positive benefits of BDNF on dendrite architecture are activity-dependent,^74^ and thus, the maintenance of normal neuronal metabolism is a critical consideration. Other therapeutics, such as the neurosteroid ganaxalone, growth hormone, glutathione, and antioxidant treatments such as melatonin, have all received attention in preclinical models of hippocampal compromise and demonstrate good promise for improved outcomes in hippocampal morphology.^30,39,45,56,76,77^

One final consideration is whether male and female offspring show a differential response for both hippocampal development and response to perinatal compromise. Sex-specific differences are shown in clinical studies of pregnancy complications, including prematurity and FGR, where males have an increased risk of mortality, morbidity, and adverse neurological outcomes.^78–83^ Sex-specific differences in placentation and fetal growth trajectory may contribute to increased vulnerability in males, with males generally having accelerated growth rates and increased growth outcomes relative to females. This accelerated growth tends to leave male fetuses vulnerable to *in utero* compromise, including metabolic disturbances and increased risk for FGR.^84,85^ Many preclinical studies included in this review did not explore the influence of sex on study outcomes. However, of the four studies that did, their findings were contradictory. Two studies in FGR mice found female mice had reduced hippocampal myelination and poorer performance in learning and memory tasks compared to males.^28,29^ Conversely, in two FGR guinea pig studies, males had poorer neurodevelopmental outcomes with reduced myelination and increased markers of hypoxia (hypoxyprobe-1, HP-1) in hippocampal tissue.^2,86^ Contrasting evidence found across these FGR studies may lie in the different models used and the age of the animals at assessment. The two mice studies were conducted on postnatal age mice after inducing FGR from embryonic day 12 with an osmotic pump infusion of TXA.^28,29^ In contrast, the guinea pig studies utilised two different models of FGR- maternal undernourishment and prenatal stress and tissue analysis was conducted at day 60 of gestation (term ~68 days), a period where males may be more susceptible to a poor *in utero* environment, compared to females.^2,86^ These results emphasise the importance of including both sexes when examining the impact of perinatal compromise.

## Conclusion

In this review, we examined the preclinical literature to reveal how hippocampal form and function are altered in response to FGR, preterm birth, intrauterine inflammation, and acute hypoxic-ischaemic insult at birth. The most striking finding that is consistent across the clinical^5^ and preclinical literature is reduced hippocampal volume. In the most severe perinatal insults, reduced hippocampal volume occurs secondary to neuronal cell loss, but more commonly, neuronal cell number is maintained but there is a disruption to dendrite outgrowth. Perinatal complications that affect brain development are most common during the 3^rd^ trimester of human pregnancy, corresponding to the period of hippocampal growth spurt. This concomitant timing of peak hippocampal arborisation and perinatal insult has profoundly adverse effects on neuronal morphology, hippocampal connectivity and functions regulated by the hippocampus, including cognition, capacity for learning and memory, emotions, and behaviours. In response to perinatal compromise, the susceptibility of hippocampal neurons is greater than for other grey matter neuron-rich regions, such as the cortex, thalamus, and brainstem. Within the *hippocampal formation*, subregion differences exist in cell populations and functions, and a number of studies have shown that the CA3 neurons are the most highly vulnerable to insult. Finally, we note that the highly susceptible nature of hippocampal neurons to damage is contributed by this region being neuron-dense and metabolically dynamic but with a relatively low vascular density. Moving forward, preclinical studies should examine neuroprotective therapies that mitigate the potential mechanisms of hippocampal cell injury, including a reduction in growth factor support, neuroinflammation and oxidative stress, incorporating assessments of both hippocampal structure and function.

## Data Availability

Data sharing is not applicable as no datasets were generated or analysed for this review.
